# A multivariate dataset on profiling personality traits, social and cognitive determinants of omani students’ entrepreneurial intention

**DOI:** 10.1016/j.dib.2023.109363

**Published:** 2023-07-01

**Authors:** Imran Saleem, Imran Anwar

**Affiliations:** a University of Buraimi, Buraimi, Oman; b University Centre for Research and Development, Chandigarh University, Mohali, India

**Keywords:** Personality traits, Opportunity recognition, Social capital, Entrepreneurial intention, Entrepreneurship education, Nascent entrepreneurs

## Abstract

This data article collects and validates a multivariate dataset on personality traits, social, and cognitive predictors of the entrepreneurial intention of 276 students of three Omani universities. Administering a validated questionnaire, a pilot study was conducted by taking a sample of 60 responses to ensure the robustness of the survey scales. Following the pilot study, the final data were collected from 276 UG and PG level students in February 2021, which were first cleaned for missing, unengaged, and outlier responses before going ahead with statistical analyses. The data were also checked for common method bias by applying Cook's distance method which was followed by establishing the measurement model (ensuring model fitness, convergent and divergent validities) by running a CFA model in AMOS. The dataset from this data article would be of significant use for the researchers studying nascent and student entrepreneurship and Oman universities and the government in developing an entrepreneurship course curriculum.

Specifications table

**Table:** 

Subject	Entrepreneurship

Specific subject area	Entrepreneurial intention, entrepreneurship education, entrepreneurial motivation, fear of failure, psychological and contextual predictors of entrepreneurial intention
Type of data	.csv Excel file Table
How the data were acquired	The data were gathered using convenience sampling from three distinct universities in the Sultanate of Oman: University of Buraimi in Buraimi, Sohar University in Sohar, and Dhofar University in Salalah. In February 2021, the UG and PG students with backgrounds in business and management were approached using their institutional emails and invited to participate in the survey. The invitation email contained a brief introduction about the study and a Google Forms link to the survey.
Data format	Raw Analyzed
Description of data collection	A pilot survey was conducted to ensure the robustness of the questionnaire. For piloting, we took a total of 60 responses and checked the internal consistency of the questionnaire scales. After the pilot survey, we conducted the final survey approaching a total of 390 students through their institutional email. An invitation email was sent to each student, which contained a brief introduction about the study along with a consent form and a Google Forms link to the survey. A total of 304 students filled out the survey (at a retrieval rate of 77.95%).
Data source location	Institution: University of Buraimi; Sohar University; Dhofar University City/Town/Region: Al-Buraimi; Sohar; Salalah Country: Oman
Data accessibility	Anwar, Imran (2023), “A Multivariate Dataset on Profiling Personality Traits, Social and Cognitive Determinants of Omani Students’ Entrepreneurial Intention”, Mendeley Data, V1, DOI: https://doi/org/10.17632/36snsp9dgp.1 URL Link: https://data.mendeley.com/datasets/36snsp9dgp/1

## Value of the Data

• This data article provides the unique primary survey-based data of business students at university level to understand the causal relationships between personality traits (innovativeness, locus of control, risk-taking propensity), social (social capital and subjective social norm), and cognition-based factors (entrepreneurial attitude, opportunity recognition, entrepreneurship education, and perceived behavior control) and the entrepreneurial intention of university students from Oman.
• The uniqueness of the dataset lies in the inclusion of personality traits, social capital, and opportunity recognition as the latent constructs in the study, as the literature suggests that the identification of a business opportunity and having social ties with existing established entrepreneurs (social capital) are found to be linked with the personality traits of nascent entrepreneurs.
• Corresponding to the problems associated with the declining TEA rate and EBO rate of Oman, these data can be of immense use for researchers from the domain of nascent/student entrepreneurship as it contains data on personality traits and socio-cognitive and psychological predictors of entrepreneurial intention.
• The results obtained from this dataset might help institutions and the government of Oman in creating an entrepreneurship course curriculum and encouraging entrepreneurship among college students.
• Considering the metric, multivariate, and latent nature of the dataset, it offers multifold possibilities for applying complex modeling like moderated mediation, serial mediation, and moderated moderation to further unfold the conditional interplay between traits, social, cognitive factors and entrepreneurial intention among university business students.
• The dataset also offers the possibility of checking the moderation of gender and entrepreneurial inclination as the data is almost equally distributed across the categories of gender (male and female) and entrepreneurial inclination (inclined and not inclined students).

## 1 Objective

Entrepreneurship has become one of the most discussed career opportunities among the younger population. Governments and educational establishments are exerting a growing focus on entrepreneurship promotion. Most individuals have entrepreneurial aspirations, but prior research indicates that just a fraction really pursue such ambitions. Understanding the variables that lead to entrepreneurial ambitions and how these factors may transform such goals into behavior is becoming more essential. Not only do inner traits and cognition forces affect the through-process of the entrepreneurs, but the surrounding environment and physical infrastructure also play a pivotal role in nurturing and augmenting individuals’ behavioral intention. As per Global Entrepreneurship Monitor Report, despite introducing several entrepreneurship support and incubation programs, Oman's total early-stage entrepreneurship (TEA rate) has been witnessed on a decline (from 16% in 2020 to 12.7% in 2021), while Oman's established business ownership rate (EBO rate) has been found as the second lowest among 46 sampled nations. Considering the fact that nascent Omani entrepreneurs are striving to transform their entrepreneurial proclivity into intention, this data article collects survey-based primary data on personality traits, social and cognitive factors, and the entrepreneurial intention of Omani university students.

## 2 Data Description

This data article collects and validates a multivariate dataset on personality traits (innovativeness, locus of control, and risk-taking propensity), social (social capital and subjective social norm), and cognition-based factors (entrepreneurial attitude, opportunity recognition, entrepreneurship education, and perceived behavior control) and the entrepreneurial intention of the students of three Omani universities. For this purpose, UG and PG level students enrolled in business and management programs at three Omani universities (viz. University of Buraimi, Sohar University, and Dhofar University) were selected as the study's target sample based on previous research in the fields of nascent/student entrepreneurship, and entrepreneurship education-intention link [1]. The rationale for selecting university level business students as the study's sample was their engagement in learning entrepreneurship education which could help them enrich their entrepreneurial mindset and form stronger entrepreneurial intention. Moreover, they also embody a vibrant segment of the population, serving as a significant wellspring of entrepreneurial talent. For data collection, a questionnaire with two parts and 42 manifest variables was constructed. Using multiple items measurement scales, the first section was created to assess ten latent constructs, including "entrepreneurial attitude," "opportunity recognition," "entrepreneurship education," "perceived behavioral control," "entrepreneurial intention," "social capital," "subjective social norm," "innovativeness," "locus of control," and "risk taking propensity." Age, gender, education level, and entrepreneurial inclination were the demographic characteristics to be recorded in the second section of the survey.

A pilot study was conducted with a sample of 60 students (20 students from each university) to assess the internal consistency and reliability of the measuring scales (using Alpha reliability) followed by conducting the final survey in February 2021 by administering a total of 390 questionnaires (130 at each university). UG and PG level students with backgrounds in business and management were approached using their institutional emails and invited to participate in the survey. The invitation email contained a brief introduction about the study along with a consent form and a Google Forms link to the survey. A total of 304 completed questionnaires (at a retrieval rate of 77.95%) out of 390 distributed questionnaires.

However, before moving further with statistical validation tests, the sample of 304 responses was vetted and cleaned since it included some disengaged and outlier responses. First, the data were checked for incomplete and incorrect answers. It was discovered that 16 respondents did not get engaged with the questionnaire; thus, their responses were removed from the sample. Second, statistical outliers in the sample data were also found using Cook's distance procedure. An outlier is a response with Cook's statistics >1 [2]. Cook's statistics >1 were used to identify 12 replies as significant statistical outliers, and they were eliminated from the analysis, leaving a final sample of 276 responses (refer to Table 1 for respondents’ demographic properties).

**Table 1:** Demographic profile of the respondents (N = 276).

Variable Name	Category	Frequency (N)	Percentage (%)
Age	Less than 22 years	143	51.8
	22 to 25 Years	36	13.0
	Above 25 years	97	35.1
Gender	Male	143	51.8
	Female	133	48.2
Level of education	UG	178	64.5
	PG	98	35.5
Entrepreneurial inclination	Inclined	111	40.2
	Not inclined	165	59.8
University	University of Buraimi, Buraimi, Oman	98	35.5
	Sohar University, Sohar, Oman	91	33.0
	Dhofar University, Salalah, Oman	87	31.5

Finally, Podsakoff and Organ [3] Harman's single factor technique was used to examine the data for statistical method bias. To account for the total explained variance, all 42 manifest variables were made to load on a single component (using “Principal Component Analysis” for extraction and “Varimax” for rotation). The findings demonstrated that all 42 manifest variables could account for a variation of 33.195%, which is far below the 50% threshold and demonstrates no method bias in the data.

A covariance-based CFA model, run in AMOS v.24, validated the measurement model's global fit indices, validities (convergence and divergence), and reliability (scale's internal consistency). Model fit indices and CFA loadings were obtained by running a CFA measurement model allowing 42 manifest variables to converge with their respective latent constructs (ten latent constructs).

The CFA model findings indicated that the model had a very good fit, with model fit indices falling into the acceptable range (refer to Table 2). The convergence of observed items with their respective latent constructs was determined by ensuring that average CFA loading for each latent construct is greater than .708 and hence accounting to an explanation of the majority (50%) of extracted variance (AVE). Convergent validity is considered to be met when the AVE value is greater than .50, which is the squared value of a latent construct's average CFA loading [4,5]. The findings, reported in Table 3, reveal that the AVE values are considerably over the threshold, assuring the data's convergent validity. Furthermore, we also ensured the internal consistency of the measurement scales by applying Cronbach's Alpha (α) and Composite Reliability (CR). If α and CR statistics are more than .70, a construct is regarded to fulfill scale reliability standards [5]. Statistics for α and CR are found to be significantly above the .70 level, satisfying the scale reliability criterion.

**Table 2:** CFA model fit indices, convergent validity, composite and Cronbach's alpha reliability.

Model	CMIN/DF	GFI	CFI	TLI	RMSEA
Study model	1.971	0.911	0.941	0.934	0.059
Recommended value	Acceptable 1-4	>0.90	>0.90	>0.90	<0.07
	Wheaton, Muthen, Alwin and Summers [6]	Shevlin and Miles [7]	Shevlin and Miles [7]	Hu and Bentler [8]	MacCallum, Browne and Sugawara [9]
Construct name with measurement item	Item loading	Avg CFA Loading	AVE	CR	Cronbach's alpha (α)
					
Entrepreneurial intention		0.801	0.642	0.911	0.904
*EI1*	*0.815*				
*EI2*	*0.697*				
*EI3*	*0.834*				
*EI4*	*0.799*				
*EI5*	*0.842*				
*EI6*	*0.819*				
Perceived behavioral control		0.834	0.695	0.874	0.870
*PBC1*	*0.773*				
*PBC2*	*0.817*				
*PBC3*	*0.911*				
Entrepreneurial attitude		0.824	0.679	0.914	0.909
*ATT1*	*0.829*				
*ATT2*	*0.739*				
*ATT3*	*0.845*				
*ATT4*	*0.858*				
*ATT5*	*0.849*				
Subjective social norm		0.770	0.593	0.831	0.826
*SSN1*	*0.739*				
*SSN2*	*0.796*				
*SSN3*	*0.775*				
Opportunity recognition		0.780	0.609	0.886	0.882
*OR1*	*0.782*				
*OR2*	*0.788*				
*OR3*	*0.759*				
*OR4*	*0.798*				
*OR5*	*0.775*				
Social capital		0.768	0.590	0.782	0.774
*SC1*	*0.748*				
*SC2*	*0.812*				
*SC3*	*0.744*				
Entrepreneurship education		0.854	0.729	0.910	0.903
*EE1*	*0.818*				
*EE2*	*0.831*				
*EE3*	*0.869*				
*EE4*	*0.878*				
*EE5*	*0.872*				
Innovativeness		0.785	0.616	0.866	0.863
*INNOV1*	*0.748*				
*INNOV2*	*0.747*				
*INNOV3*	*0.842*				
*INNOV4*	*0.803*				
Locus of control		0.778	0.602	0.889	0.886
*LOC1*	*0.738*				
*LOC2*	*0.702*				
*LOC3*	*0.771*				
*LOC4*	*0.832*				
*LOC5*	*0.848*				
Risk taking propensity		0.796	0.634	0.840	0.834
*RTP1*	*0.872*				
*RTP2*	*0.804*				
*RTP4*	*0.712*

**Table 3:** Correlations, divergent validity, and descriptive statistics.

Construct name	EI	PBC	EA	SSN	OR	SC	EE	INN	LoC	RTP
EI	.801									
PBC	.516^⁎⁎^	.834								
EA	.648^⁎⁎^	.503^⁎⁎^	.824							
SSN	.453^⁎⁎^	.245^⁎⁎^	.479^⁎⁎^	.770						
OR	.647^⁎⁎^	.568^⁎⁎^	.677^⁎⁎^	.408^⁎⁎^	.780					
SC	.337^⁎⁎^	.357^⁎⁎^	.314^⁎⁎^	.317^⁎⁎^	.520^⁎⁎^	.768				
EE	.671^⁎⁎^	.591^⁎⁎^	.650^⁎⁎^	.414^⁎⁎^	.678^⁎⁎^	.403^⁎⁎^	.854			
INN	.589^⁎⁎^	.573^⁎⁎^	.649^⁎⁎^	.409^⁎⁎^	.616^⁎⁎^	.461^⁎⁎^	.656^⁎⁎^	.785		
LoC	.585^⁎⁎^	.487^⁎⁎^	.564^⁎⁎^	.425^⁎⁎^	.595^⁎⁎^	.309^⁎⁎^	.638^⁎⁎^	.619^⁎⁎^	.776	
RTP	.523^⁎⁎^	.427^⁎⁎^	.423^⁎⁎^	.308^⁎⁎^	.581^⁎⁎^	.307^⁎⁎^	.526^⁎⁎^	.514^⁎⁎^	.633^⁎⁎^	.796
Mean	5.805	4.829	5.809	5.263	5.207	4.296	5.392	5.157	5.652	5.249
SD	1.086	1.217	1.081	1.285	1.081	1.423	1.118	1.141	1.115	1.271
Skewness	-0.999	-0.221	-1.036	-0.673	-0.226	-0.170	-0.436	-0.419	-0.886	-0.605

Note: Squared root of AVE has been shown in bold on diagonals, and it should be greater than off-diagonal values for divergent validity. EI = Entrepreneurial Intention; PBC = Perceived Behavioral Control; EA = Entrepreneurial Attitude; SSN = Subjective Social Norm; OR = Opportunity Recognition; SC = Social Capital; EE = Entrepreneurship Education; INN = Innovativeness; LoC = Locus of Control; RTP = Risk Taking Propensity

In line with Fornell and Larcker [10] approach for ensuring discriminant validity, we compared the squared root value of AVE (bold diagonal values) of each construct to below-diagonal values (bivariate correlations) and found that the bold values on diagonals are greater than inter-construct bivariate correlations. The excess of the squared root of AVE over bivariate correlations infers to the fulfillment of the discriminant validity criterion [10]. Moreover, we also assessed the discriminant validity using the HTMT ratio criterion (see Table 4). Discriminant validity tends to be established if HTMT ratios among the latent variables are found to be < .85 Kline [11]. It is evident from Table 4 that HTMT ratios are less than the threshold of .85; hence measurement model meets discriminant validity criteria following the HTMT approach as well. In order to support the multivariate normality assumption, Table 3 additionally provides skewness statistics along with descriptive statistics (mean and SD) for each latent variable. The range of the skewness statistics for each latent variable is between -2 and +2, indicating that the data are normal [11].

**Table 4:** HTMT ratio analysis for discriminant validity.

Construct name	EI	PBC	EA	SSN	OR	SC	EE	INN	LoC	RTP
EI										
PBC	.564									
EA	.662	.730								
SSN	.575	.715	.661							
OR	.363	.619	.597	.536						
SC	.644	.650	.688	.718	.568					
EE	.439	.372	.564	.398	.510	.626				
INN	.656	.710	.733	.622	.527	.644	.474			
LoC	.555	.628	.706	.651	.518	.668	.373	.701		
RTP	.499	.487	.604	.601	.394	.674	.383	.597	.736	

Note: EI = Entrepreneurial Intention; PBC = Perceived Behavioral Control; EA = Entrepreneurial Attitude; SSN = Subjective Social Norm; OR = Opportunity Recognition; SC = Social Capital; EE = Entrepreneurship Education; INN = Innovativeness; LoC = Locus of Control; RTP = Risk Taking Propensity

## 3 Experimental Design, Materials and Methods

The questionnaire was created using validated measurement scales borrowed from published studies. Measurement scales for entrepreneurial attitude, perceived behavioral control, and entrepreneurial intention were taken from Liñán and Chen [12]. For subjective social norm and social capital, Trivedi [13] and Liao and Welsch [14] were duly cited, respectively. Further, for measuring opportunity recognition, we borrowed a scale from Ozgen and Baron [15]. We cited Lorz [16] for entrepreneurship education scale. Lastly, for capturing three personality traits viz. innovativeness, locus of control, and risk-taking propensity, we adopted the scales developed by Jackson [17], Levenson [18], Chye Koh [19], respectively.

The questionnaire items were first subjected to screening for subjectivity and linguistic correctness checks before going forward with the pilot and main survey. Four university professors/academicians with experience in the teaching and study of entrepreneurship received the questionnaire to assess its quality and linguistic correctness. To strengthen the validity of the questionnaire, their recommendations about the measurement scales' subjectivity and unidimensionality were considered. In accordance with the qualitative remedial recommendations of Podsakoff, MacKenzie, Lee and Podsakoff [20], the wording of each questionnaire item was also made plain, straightforward, single-faceted, and error-free, eliminating double-barreled questions. The survey's goal was explained in the questionnaire's introduction, and the respondents were also assured that their answers would be kept anonymous and private. However, the poll did not request any information that may allow for personal identification, such as name, address, email, etc.

Following the positivist research approach and cross-sectional design, the main survey was undertaken after the pilot survey, and data were gathered for both predictors and the outcome variable at the same time using convenience sampling.

## Ethics Statements

Data were anonymized, even no personal information like contact numbers or/and email addresses were solicited; therefore, the Research Ethics Committee of the University of Buraimi exempted this study from ethical approval. Informed consent was obtained from all individual participants included in the study. Their participation was voluntary, and they could withdraw from the study at any point. No minor participants were recruited for the survey.

## CRediT authorship contribution statement

**Imran Saleem:** Conceptualization, Data curation, Writing – original draft, Writing – review & editing. **Imran Anwar:** Conceptualization, Writing – original draft, Data curation, Methodology, Writing – review & editing.

## Declaration of Competing Interest

On behalf of all authors, the corresponding author states that there is no conflict of interest.

## Data Availability

The Dataset on Personality Traits, Social Capital, and Cognitive Predictors of Omani Students' Entrepreneurial Intention (Original data) (Mendeley Data).
